# Incidence and Factors Associated With In-Hospital Acute Myocardial Infarction in Patients With Obesity Undergoing Transcatheter Aortic Valve Replacement: A National Inpatient Sample Analysis

**DOI:** 10.14740/cr2257

**Published:** 2026-08-31

**Authors:** Li Li Zheng, Gui Zhen Xu, Xiao Jun Zeng, Zi Hao Zhou, Hao Xie, Tao Shi, Long Hua Liu, Yan Zhu, Xiang Hua Cao, Wen Gui Liu, Sheng Qi Xie

**Affiliations:** a Department of Anesthesiology, Dongguan Songshan Lake Tungwah Hospital, Dongguan, Guangdong 523000, China; b Kangda College of Nanjing Medical University, Nanjing, Jiangsu 210000, China; c Division of Orthopaedic Surgery, Department of Orthopaedics, Nanfang Hospital, Southern Medical University, Guangzhou, Guangdong 510515, China; d Department of Anesthesiology, Dongguan Tungwah Hospital, Dongguan, Guangdong, China; e These authors contributed equally to this work.

**Keywords:** Transcatheter aortic valve replacement, Acute myocardial infarction, Patients with obesity, Risk factors, In-hospital outcomes

## Abstract

**Background:**

The impact of obesity on acute myocardial infarction (AMI) risk in patients undergoing transcatheter aortic valve replacement (TAVR) remains poorly characterized, particularly with respect to the incidence and specific factors associated with this complication. This study aimed to determine the incidence, associated factors, and clinical implications of in-hospital AMI among patients with obesity undergoing TAVR.

**Methods:**

We analyzed the National Inpatient Sample (2016–2022) to identify adult patients with obesity (body mass index (BMI) ≥ 30 kg/m^2^) undergoing TAVR, with non-obese patients as a comparative cohort. Multivariable logistic regression identified factors associated with in-hospital AMI.

**Results:**

Among 19,971 patients with obesity undergoing TAVR, 506 (2.5%) experienced in-hospital AMI, compared with 2.7% among non-obese patients (P = 0.295). Patients with obesity were younger (median age 75 vs. 81 years, P < 0.001) and had lower in-hospital mortality (0.8% vs. 1.3%, P = 0.002) but longer hospital stays (3 vs. 2 days, P < 0.001) and higher hospitalization costs ($217,785 vs. $179,887, P < 0.001) compared with patients without obesity. Factors associated with higher AMI odds in the obesity cohort included medium hospital size (odds ratio (OR) 1.687, 95% confidence interval (CI) 1.086–2.619), large hospital size (OR 1.840, 95% CI 1.234–2.744), deficiency anemia (OR 1.466, 95% CI 1.196–1.796), chronic blood loss anemia (OR 2.960, 95% CI 1.604–5.463), congestive heart failure (OR 2.530, 95% CI 1.871–3.421), fluid and electrolyte disorders (OR 2.734, 95% CI 2.239–3.338), other neurological disorders (OR 2.092, 95% CI 1.453–3.011), peripheral vascular disorders (OR 1.260, 95% CI 1.017–1.561), pulmonary circulation disorders (OR 1.297, 95% CI 1.054–1.595), and weight loss (OR 2.156, 95% CI 1.400–3.319). Conversely, elective admission (OR 0.046, 95% CI 0.037–0.058) was associated with lower AMI odds. AMI was associated with higher mortality (3.8% vs. 0.8%), prolonged hospitalization (11 vs. 3 days), and increased costs ($349,531 vs. $217,785) (all P < 0.001).

**Conclusions:**

In-hospital AMI occurs in 2.5% of patients with obesity undergoing TAVR—a rate comparable to that of patients without obesity (2.7%, P = 0.295)—and is associated with worse outcomes. Despite lower overall mortality in the obesity cohort—consistent with the obesity paradox—AMI risk was not similarly attenuated, suggesting that the protective effect of obesity does not uniformly extend to all periprocedural complications. Rather than simply documenting an expected complication rate, the clinical value of our study lies in identifying potentially modifiable factors associated with AMI specifically in the obesity cohort—including anemia, fluid and electrolyte disorders, and weight loss—that may inform targeted preoperative optimization.

## Introduction

Transcatheter aortic valve replacement (TAVR) has emerged as a primary treatment alternative for patients with symptomatic severe aortic stenosis, particularly in those at intermediate to high surgical risk [1, 2], with its indications expanding to increasingly lower-risk populations [3]. As procedural techniques evolve, TAVR volumes continue to rise globally [4]. However, acute myocardial infarction (AMI) remains a serious complication in patients undergoing TAVR, primarily resulting from coronary embolism of dislodged calcific debris or valve frame compression of the coronary ostia [5]. The clinical significance of periprocedural AMI is underscored by meta-analyses demonstrating its strong association with 30-day (odds ratio (OR) 8.5) and mid-term mortality (OR 6.5) [6], with one study reporting periprocedural myocardial injury in 20.5% of patients [7]. Identifying factors associated with in-hospital AMI is therefore critical for optimizing perioperative management.

This topic is particularly relevant in the context of the global obesity epidemic, as the proportion of obese patients undergoing TAVR has increased substantially. While obesity is associated with an increased prevalence of cardiovascular risk factors, an “obesity paradox” has been described, wherein obese patients exhibit lower post-TAVR mortality than their normal-weight counterparts [8]. However, this paradigm has recently been challenged by evidence demonstrating comparable outcomes across body mass index (BMI) categories [9] and, conversely, significantly higher risks for patients with super obesity [10]. These seemingly contradictory findings highlight the heterogeneous impact of obesity on TAVR outcomes, suggesting that risk profiles may differ by complication type.

Despite these insights, the incidence and specific factors associated with AMI among patients with obesity undergoing TAVR remain inadequately characterized, even though obesity is a well-established risk factor for AMI in the general population. Existing studies have largely focused on all-cause mortality or composite endpoints (e.g., death, stroke, and bleeding), leaving the detailed profile of AMI—particularly its non-traditional associated factors such as anemia, electrolyte disorders, and weight loss—underexplored in this growing patient subset. These knowledge gaps justify a dedicated analysis focused specifically on AMI in this population. To address this gap, we conducted a retrospective analysis of the National Inpatient Sample (NIS) database, focusing on patients with obesity who underwent TAVR from 2016 to 2022, with patients without obesity as a comparative cohort. Specifically, we aimed to: (1) determine the incidence of in-hospital AMI in this cohort; (2) identify factors independently associated with in-hospital AMI among patients with obesity; (3) evaluate the impact of AMI on in-hospital mortality, length of stay, and hospitalization costs; and (4) compare outcomes between obese and non-obese patients. Our objective was to describe the incidence and explore associated factors within the obese patient cohort, rather than to compare outcomes across BMI categories.

## Materials and Methods

### Data source

This study utilized the NIS database, which is maintained by the Healthcare Cost and Utilization Project (HCUP) and sponsored by the Agency for Healthcare Research and Quality (AHRQ). The NIS represents the largest publicly available all-payer inpatient database in the United States, capturing approximately 20% of all hospitalizations through a stratified sample of over 1,000 hospitals nationwide. This sampling strategy yields data on more than 7 million unweighted hospital stays annually, providing excellent generalizability to the national population [11]. The database encompasses patient demographic characteristics, hospital-level information, discharge disposition, length of stay, total hospitalization charges, and up to 40 diagnostic and procedural codes derived from the International Classification of Diseases, Clinical Modification (ICD-CM). Access details, restrictions, and direct web links for the NIS database are provided in the Data Availability statement. This study was deemed exempt from institutional review board approval by the Ethics Committee of Dongguan Songshan Lake Tungwah Hospital, as it utilized de-identified, publicly available data from the NIS database. Therefore, informed consent was not required.

### Study population and design

**Figure 1:**
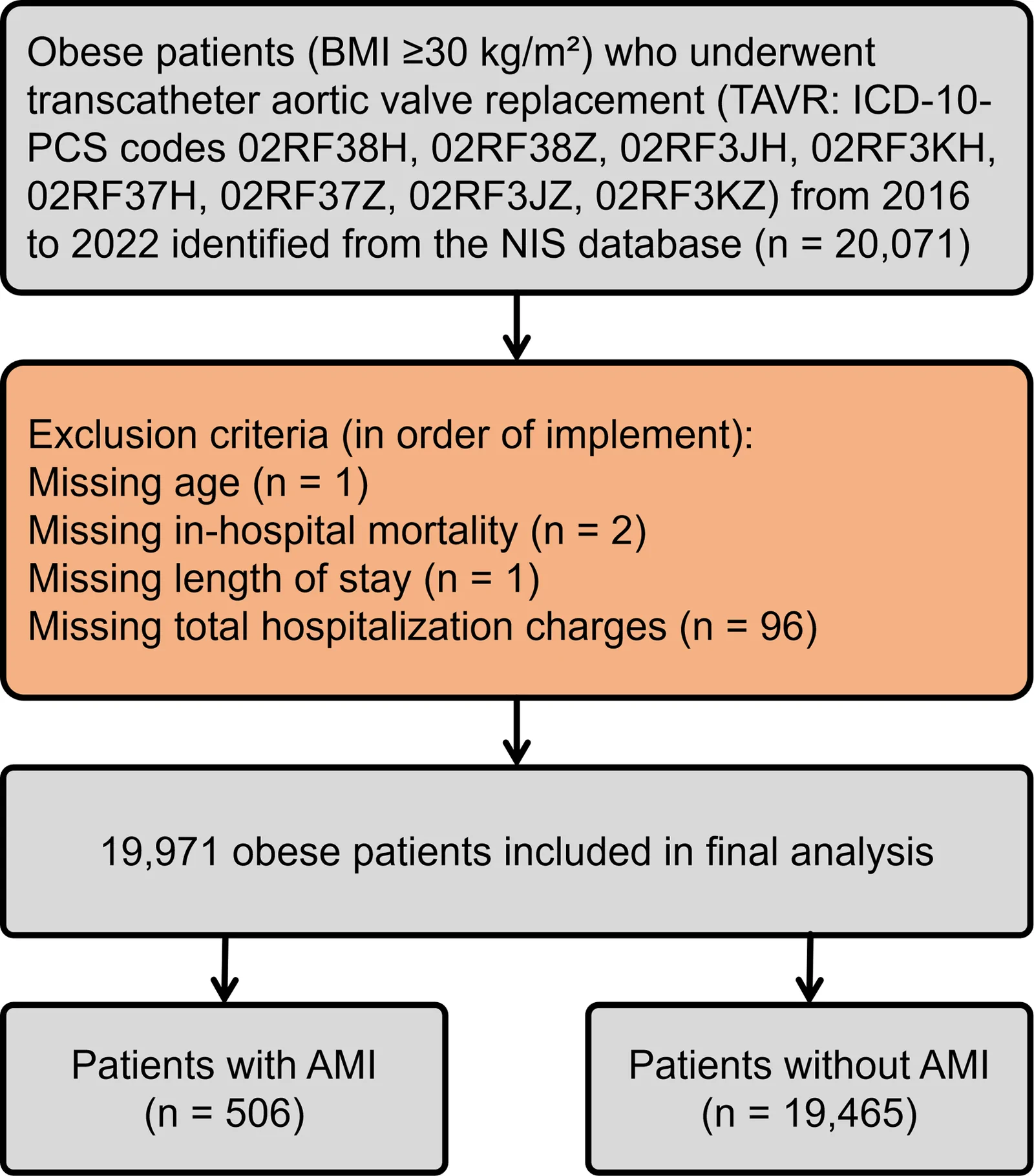
Flowchart of patient selection for in-hospital AMI among patients with obesity undergoing TAVR. AMI: acute myocardial infarction; TAVR: transcatheter aortic valve replacement.

We conducted a retrospective cohort study using the NIS database from January 2016 to December 2022. Adult patients (aged ≥ 18 years) undergoing TAVR were identified using the International Classification of Diseases, Tenth Revision, Procedure Coding System (ICD-10-PCS) codes (Supplementary Material 1, cr.elmerpub.com). Within this cohort, patients with a diagnosis of obesity were identified using ICD-10-CM codes corresponding to a BMI of 30 kg/m^2^ or greater (Supplementary Material 1, cr.elmerpub.com). The validity of these ICD-10-CM codes for identifying obesity has been previously confirmed, with high positive predictive values for BMI ≥ 30 kg/m^2^ in administrative claims data [12]. To provide a comparative context, a non-obese control cohort was identified from the same TAVR population by excluding all patients with any ICD-10-CM diagnosis codes for obesity (E66.-) or BMI codes of 30 kg/m^2^ or greater (Z68.30–Z68.39 and Z68.41–Z68.45). Thus, the two groups were mutually exclusive: patients with any obesity-related ICD codes were assigned to the obesity cohort, while those without any such codes were assigned to the non-obese cohort. Baseline characteristics, AMI incidence, in-hospital mortality, length of stay, and total hospitalization charges were compared between the two groups. Patients with missing data on age, sex, or in-hospital mortality were excluded from the analysis (Fig. 1). The study population was subsequently divided into two groups based on the occurrence of AMI during the index hospitalization. AMI was identified using ICD-10-CM codes (Supplementary Material 1, cr.elmerpub.com). Patients without documented AMI served as the reference group. All coding algorithms were previously validated, with detailed code lists and corresponding references provided in Supplementary Material 1 (cr.elmerpub.com).

### Variables and outcomes

We extracted comprehensive patient demographic and hospital characteristics as detailed in Table 1. Preoperative comorbidities were identified using ICD-10-CM codes corresponding to the Elixhauser comorbidity measures, defined according to Quan et al 2005 [13], which includes two distinct anemia-related comorbidity measures—deficiency anemia and chronic blood loss anemia—as separate coding categories. These categories were retained as defined to maintain consistency with the validated Elixhauser algorithm. Similarly, the Elixhauser category “pulmonary circulation disorders” encompasses a range of conditions including pulmonary hypertension and pulmonary embolism, and was retained as a standardized coding category without further subclassification. The number of comorbidities was categorized as < 3 and ≥ 3 for analytical purposes. The primary outcome was the incidence of in-hospital AMI among patients with obesity undergoing TAVR, defined as a diagnosis of AMI occurring during the index hospitalization, identified using ICD-10-CM codes (Supplementary Material 1, cr.elmerpub.com). As the NIS database does not provide precise timing of events relative to the procedure, this outcome encompasses AMI events that may have occurred before, during, or after TAVR. Secondary outcomes included in-hospital mortality, length of hospital stay, total hospitalization charges, and postoperative complications (sepsis, blood transfusion, acute respiratory failure, hemorrhage, cardiac arrest, arrhythmia, and pneumonia), all identified using corresponding ICD-10-CM diagnostic codes.

**Table 1:** Variables Used in Binary Logistic Regression Analysis

Variables categories	Specific variables
Patient demographics	Age (≥ 18 years), sex (male and female), race (White, Black, Hispanic, Asian or Pacific Islander, Native American and Other)
Hospital characteristics	Type of admission (non-elective, elective), bed size of hospital (small, medium, large), teaching status of hospital (nonteaching, teaching), location of hospital (rural, urban), type of insurance (Medicare, Medicaid, private insurance, self-pay, no charge, other), location of the hospital (northeast, Midwest or north central, south, west)
Comorbidities	AIDS, alcohol abuse, deficiency anemia, rheumatoid diseases, chronic blood loss anemia, congestive heart failure, chronic pulmonary disease, coagulopathy, depression, diabetes (uncomplicated), diabetes (with chronic complications), drug abuse, hypertension, liver disease, lymphoma, fluid and electrolyte disorders, metastatic cancer, neurological disorders, paralysis, peripheral vascular disorders, psychoses, pulmonary circulation disorders, renal failure, solid tumor without metastasis, peptic ulcer disease, and weight loss

AIDS: acquired immunodeficiency syndrome.

### Statistical analysis

Continuous variables were presented as median (range) and compared using the Mann–Whitney U test, while categorical variables were expressed as frequencies with percentages and compared using the Chi-square or Fisher’s exact test, as appropriate. The unit of analysis was hospitalization rather than individual patient. Statistical significance was defined as a two-sided P < 0.05. We first calculated the overall AMI incidence among obese TAVR patients and compared baseline characteristics and preoperative comorbidities between patients with and without AMI. Variables with P < 0.05 in univariate analysis were entered into multivariable logistic regression to identify factors independently associated with in-hospital AMI. Clinically relevant variables and variables selected *a priori* were also included in multivariable models. The models adjusted for age, sex, race, comorbidity burden, insurance type, hospital bed size, admission type, teaching status, hospital region, and individual preoperative comorbidities. Results were reported as adjusted ORs with 95% confidence intervals (CIs). Separate multivariable logistic regression analyses were performed to examine associations between postoperative complications and in-hospital AMI, adjusting for the same confounders. National estimates were generated using NIS discharge weights. All analyses accounted for the complex survey design of the NIS database to ensure nationally representative inferences.

## Results

### Incidence of in-hospital AMI among obese patients undergoing TAVR

**Figure 2:**
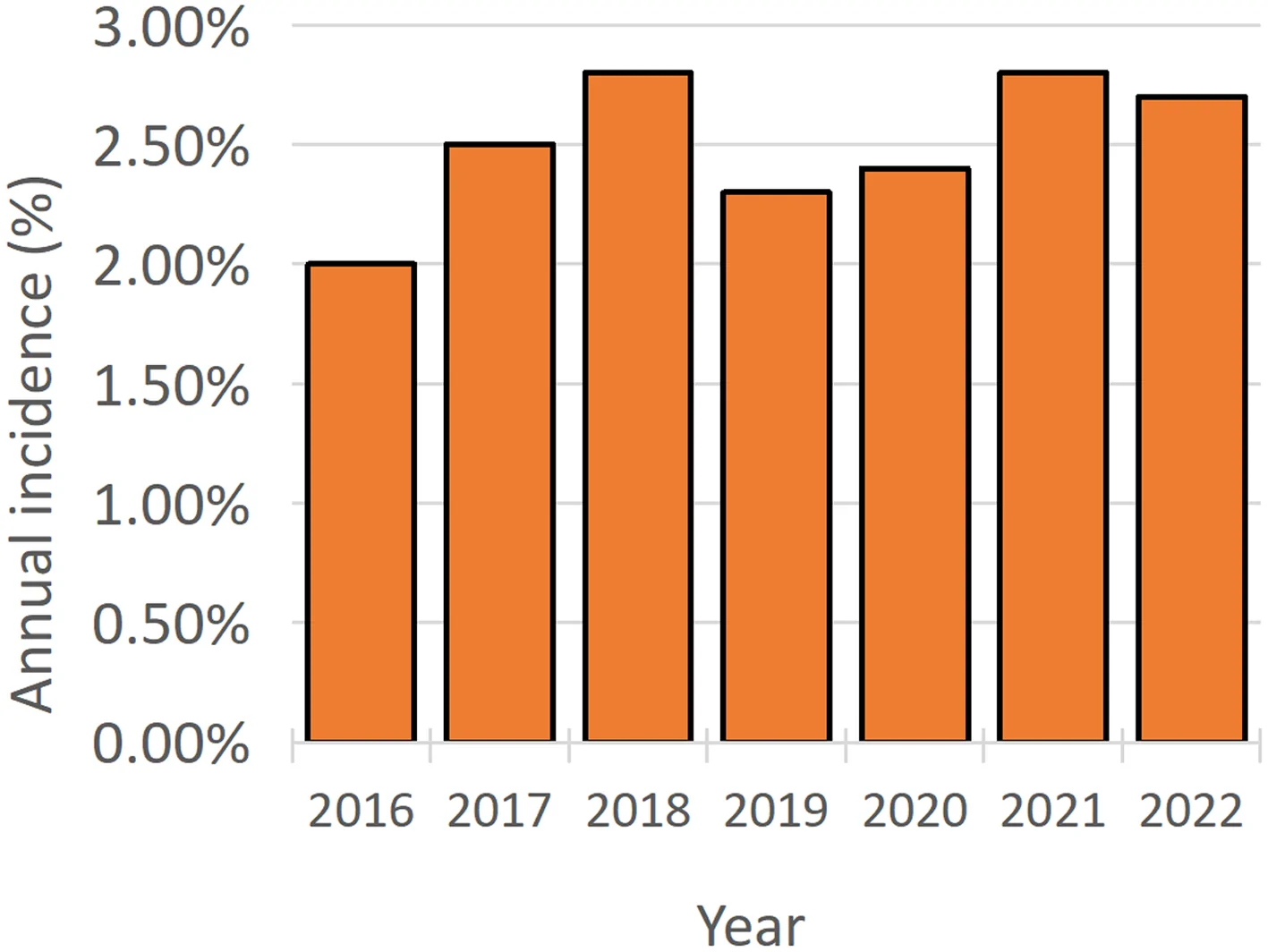
Annual incidence of in-hospital acute myocardial infarction among patients with obesity undergoing TAVR. TAVR: transcatheter aortic valve replacement.

From 2016 to 2022, a total of 19,971 (weighted N = 99,855) patients with obesity and 91,850 (weighted N = 459,250) non-obese patients undergoing TAVR were identified in the NIS database. Among patients with obesity, 506 (weighted N = 2,530) developed AMI during the index hospitalization, yielding an overall weighted incidence of 2.5%, compared with 2.7% among non-obese patients (P = 0.295) (Table 2). The annual incidence of AMI is shown in Figure 2.

**Table 2:** Patient Characteristics and Outcomes of In-Hospital AMI Among Patients With Obesity Undergoing TAVR, With Non-Obese Comparison

Characteristics	Obesity without AMI	Obesity with AMI	P-value*	Non-obese overall
Total (unweighted n, weighted N)	19,465 (weighted N = 97,325)	506 (weighted N = 2,530)	-	91,850 (weighted N = 459,250)
Total weighted incidence (%)	97.5%	2.5%	0.295	2.7%
Age (median, years)	75 (70, 81)	74 (68, 80)	0.009	81 (75, 86)
Age group (%)				
18–64	10.0	13.8	0.005	4.9
≥ 65	90.0	86.2		95.1
Gender (%)				
Male	49.9	52.6	0.235	57.8
Female	50.1	47.4		42.2
Race (%)				
White	85.3	79.4	< 0.001	84.8
Black	4.6	6.5		3.7
Hispanic	4.6	9.7		4.7
Asian or Pacific Islander	0.5	0.2		1.6
Native American	0.3	0.2		0.3
Other	4.7	4.0		4.9
Number of comorbidity (%)				
< 3	4.6	2.4	0.018	8.7
≥ 3	95.4	97.6		91.3
LOS (median, days)	3 (1,4)	11 (6,15)	< 0.001	2 (1,3)
TOTCHG (median, $)	217,785 (132,904–261,060)	349,531 (189,234–454,780)	< 0.001	179,887 (133,345–262,318)
Type of insurance (%)				
Medicare	84.1	80.6	0.330	88.4
Medicaid	2.1	4.0		1.3
Private insurance	11.1	12.5		7.6
Self-pay	0.4	1.4		0.4
No charge	0.0	0.0		0.0
Other	2.2	1.6		2.1
Bed size of hospital (%)				
Small	8.5	5.5	0.005	7.9
Medium	22.1	18.8		21.7
Large	69.4	75.7		70.4
Elective admission (%)	85.2	20.8	< 0.001	83.6
Type of hospital (teaching %)	89.4	88.7	0.658	89.4
Hospital location (%)				
Rural	1.6	2.8	0.050	1.4
Urban	98.4	97.3		98.6
Region of hospital (%)				
Northeast	20.9	23.3	0.001	22.9
Midwest or North Central	26.4	20.0		22.7
South	35.0	33.6		33.9
West	17.7	23.1		20.5
Died (%)	0.8	3.8	< 0.001	1.3

*P-value comparing obese with AMI vs. obese without AMI within the obesity cohort. All percentages are weighted. Weighted national estimates were derived using NIS discharge weights. AMI: acute myocardial infarction; TAVR: transcatheter aortic valve replacement; LOS: length of stay; NIS: National Inpatient Sample; TOTCHG: total charges.

### Comparison between obese and non-obese patients

Compared with patients without obesity, the obesity cohort was significantly younger (median age 75 vs. 81 years, P < 0.001), had a higher proportion of female patients (50.0% vs. 42.2%, P < 0.001), and had a higher comorbidity burden (≥ 3 comorbidities: 95.5% vs. 91.3%, P < 0.001). Despite their higher comorbidity burden, patients with obesity had significantly lower in-hospital mortality (0.8% vs. 1.3%, P = 0.002) but longer hospital stays (3 vs. 2 days, P < 0.001) and higher hospitalization costs ($217,785 vs. $179,887, P < 0.001) compared with non-obese patients. However, the incidence of AMI did not differ significantly between the two groups (2.5% vs. 2.7%, P = 0.295).

### Patient demographics between the two groups

Significant differences were observed in baseline characteristics between patients with and without in-hospital AMI (Table 2). Patients who experienced AMI were slightly younger (median age 74 vs. 75 years, P = 0.009). The proportion of patients aged 18–64 years was higher in the AMI group compared to the non-AMI group (13.8% vs. 10.0%, P = 0.005), while those aged ≥ 65 years were less frequent in the AMI group (86.2% vs. 90.0%, P = 0.005). No significant difference in sex distribution was found between the two groups (P = 0.235).

Racial distribution differed significantly (P < 0.001). White patients constituted a lower proportion in the AMI group (79.4% vs. 85.3%), whereas Black (6.5% vs. 4.6%) and Hispanic (9.7% vs. 4.6%) patients were overrepresented in the AMI group. The proportions of Asian or Pacific Islander, Native American, and other races were similar between groups.

Patients with AMI had a higher comorbidity burden: those with ≥ 3 comorbidities accounted for 97.6% of the AMI group compared to 95.4% of the non-AMI group (P = 0.018).

### Hospital characteristics between the two groups

Hospital characteristics also differed significantly (Table 2). Patients with AMI were more likely to be treated in large hospitals (75.7% vs. 69.4%) and less likely in small (5.5% vs. 8.5%) or medium (18.8% vs. 22.1%) hospitals (P = 0.005). Elective admission was markedly less frequent in the AMI group (20.8% vs. 85.2%, P < 0.001). Teaching hospital status did not differ between groups (P = 0.658).

Geographic variation was observed (P = 0.001). The proportion of AMI was higher in the Northeast (23.3% vs. 20.9%) and West (23.1% vs. 17.7%) regions, and lower in the Midwest or North Central (20.0% vs. 26.4%) and South (33.6% vs. 35.0%) compared to the non-AMI group. Insurance type did not show significant differences (P = 0.330).

### Adverse impact of AMI

As shown in Table 2, in-hospital mortality was significantly higher in patients with AMI (3.8% vs. 0.8%, P < 0.001). The median length of hospital stay was substantially prolonged in the AMI group (11 vs. 3 days, P < 0.001). Consequently, total hospitalization charges were markedly increased, with a median difference of $131,746 ($349,531 vs. $217,785, P < 0.001).

### Factors associated with in-hospital AMI among obese patients undergoing TAVR

**Figure 3:**
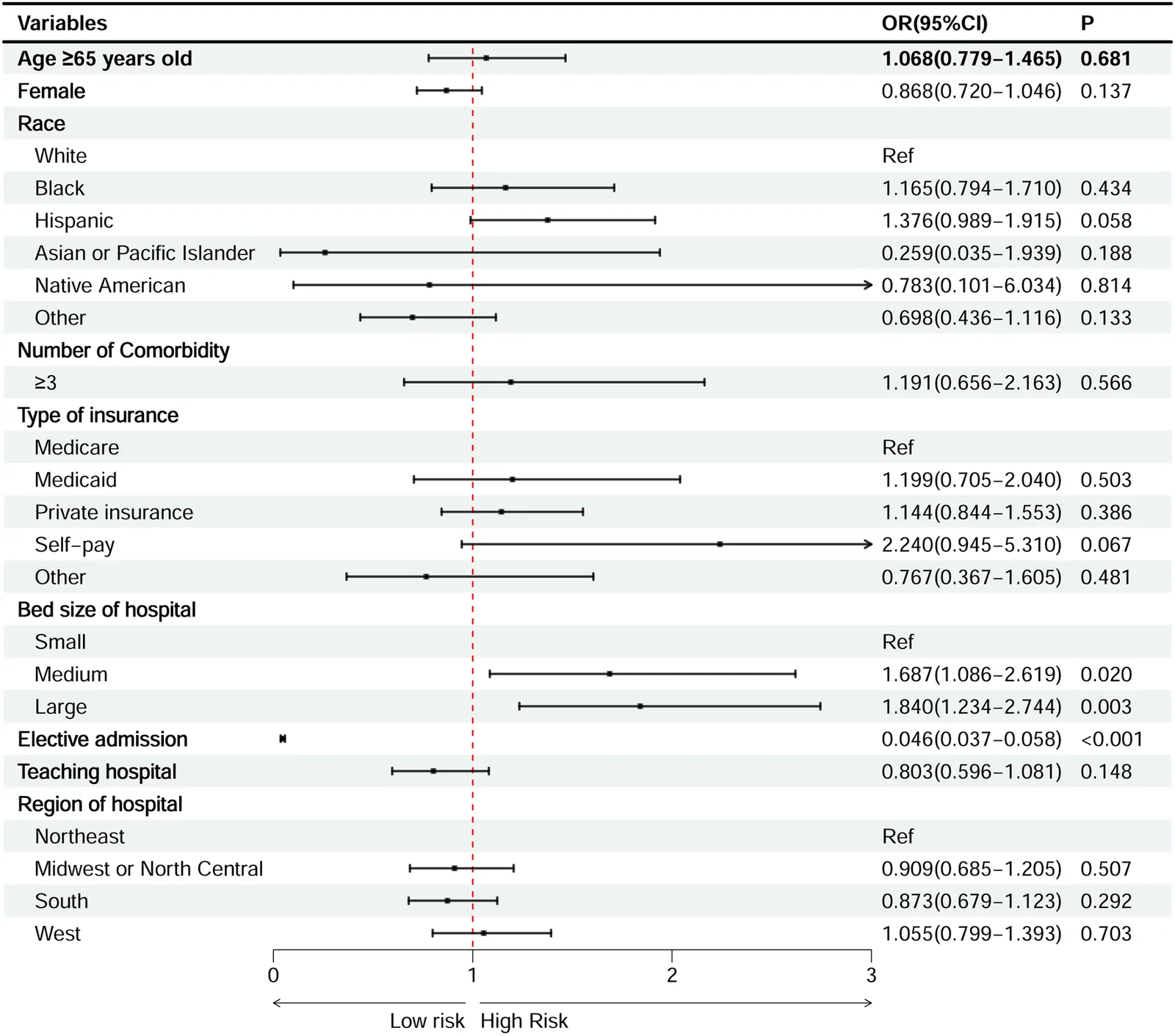
Factors associated with in-hospital AMI among patients with obesity undergoing TAVR. AMI: acute myocardial infarction; TAVR: transcatheter aortic valve replacement.

After multivariable adjustment, the following factors remained significantly associated with AMI (Fig. 3). Compared to small hospitals, medium-sized hospitals (OR = 1.687, 95% CI: 1.086–2.619, P = 0.020) and large hospitals (OR = 1.840, 95% CI: 1.234–2.744, P = 0.003) were associated with significantly increased odds of AMI. Elective admission was associated with significantly lower odds of AMI (OR = 0.046, 95% CI: 0.037–0.058, P < 0.001). Age ≥ 65 years, female sex, race categories, number of comorbidities (≥ 3), insurance types, teaching hospital status, and hospital region were not independently associated with AMI in the multivariable model (all P > 0.05).

### Preoperative comorbidities associated with in-hospital AMI among obese patients undergoing TAVR

**Figure 4:**
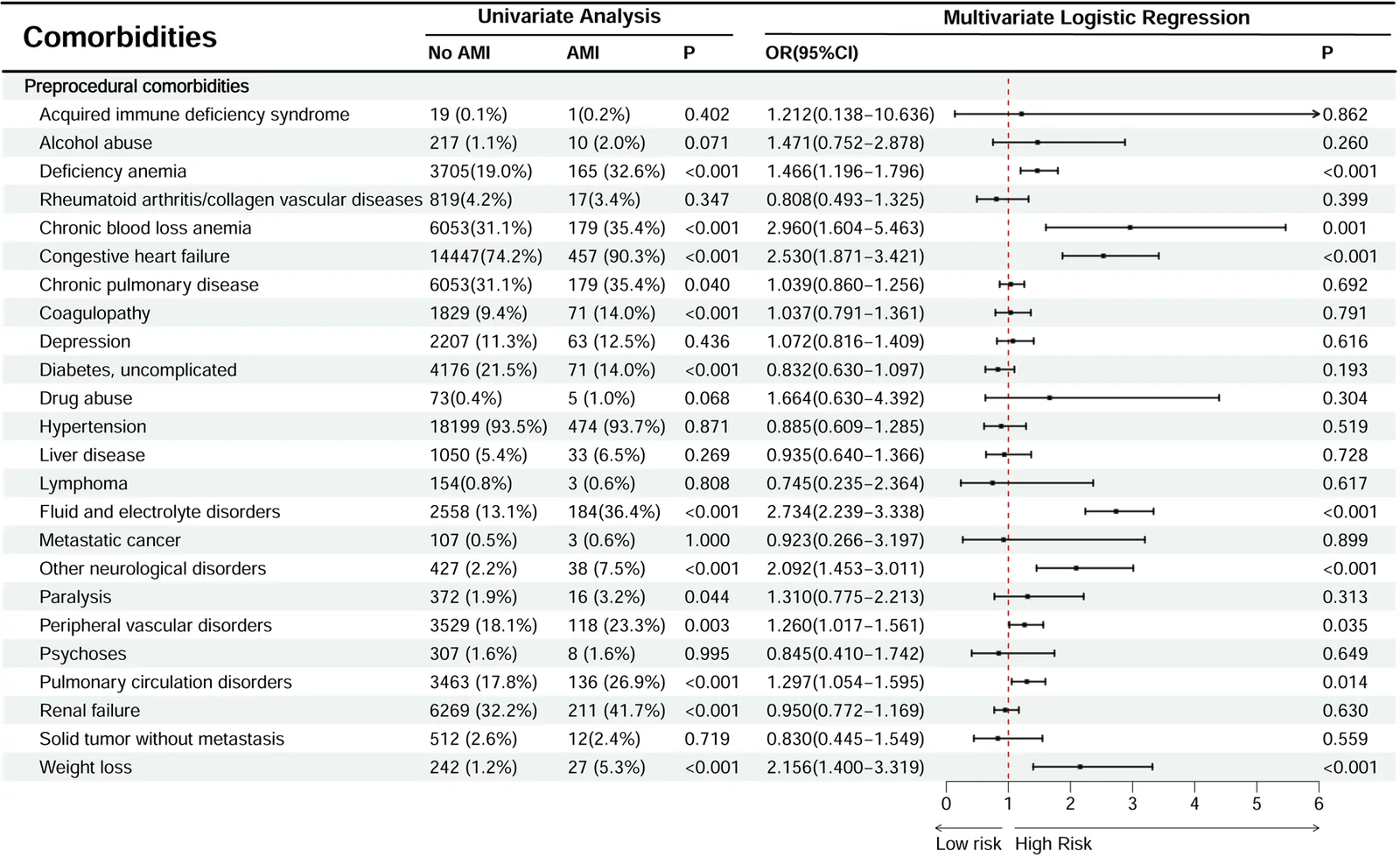
Relationship between in-hospital AMI and preoperative comorbidities among patients with obesity undergoing TAVR. AMI: acute myocardial infarction; TAVR: transcatheter aortic valve replacement.

Univariate analysis revealed multiple preoperative comorbidities significantly associated with AMI (Fig. 4). After multivariable adjustment, the following comorbidities remained significantly associated with AMI: deficiency anemia (OR = 1.466, 95% CI: 1.196–1.796, P < 0.001), chronic blood loss anemia (OR = 2.960, 95% CI: 1.604–5.463, P = 0.001), congestive heart failure (OR = 2.530, 95% CI: 1.871–3.421, P < 0.001), fluid and electrolyte disorders (OR = 2.734, 95% CI: 2.239–3.338, P < 0.001), other neurological disorders (OR = 2.092, 95% CI: 1.453–3.011, P < 0.001), peripheral vascular disorders (OR = 1.260, 95% CI: 1.017–1.561, P = 0.035), pulmonary circulation disorders (OR = 1.297, 95% CI: 1.054–1.595, P = 0.014), and weight loss (OR = 2.156, 95% CI: 1.400–3.319, P < 0.001). Other comorbidities, including acquired immune deficiency syndrome (AIDS), alcohol abuse, rheumatoid arthritis, chronic pulmonary disease, coagulopathy, depression, diabetes, drug abuse, hypertension, liver disease, lymphoma, metastatic cancer, paralysis, psychoses, renal failure, and solid tumor without metastasis, did not retain statistical significance in multivariable analysis (all P > 0.05).

### Association between in-hospital complications and in-hospital AMI among obese TAVR hospitalizations

**Figure 5:**
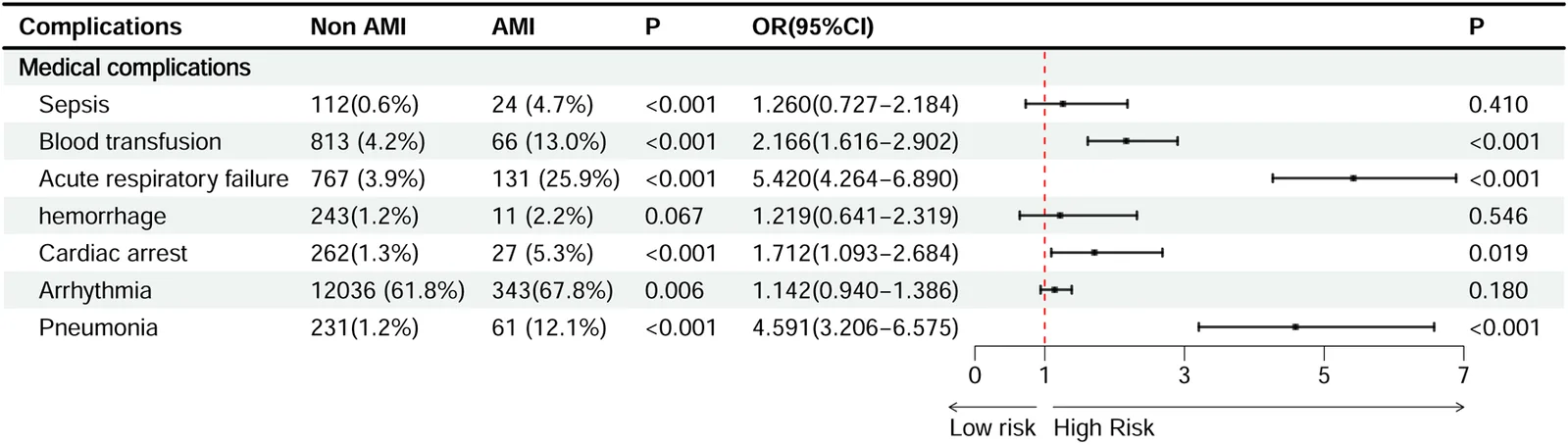
Association between in-hospital complications and AMI among patients with obesity undergoing TAVR. AMI: acute myocardial infarction; TAVR: transcatheter aortic valve replacement.

Several postoperative complications occurred more frequently in patients with AMI (Fig. 5). In univariate analysis, sepsis, blood transfusion, acute respiratory failure, cardiac arrest, arrhythmia, and pneumonia showed significantly higher incidence in the AMI group (all P < 0.001, except arrhythmia P = 0.006). Hemorrhage did not differ significantly (P = 0.067).

After multivariable adjustment, blood transfusion (OR = 2.166, 95% CI: 1.616–2.902, P < 0.001), acute respiratory failure (OR = 5.420, 95% CI: 4.264–6.890, P < 0.001), cardiac arrest (OR = 1.712, 95% CI: 1.093–2.684, P = 0.019), and pneumonia (OR = 4.591, 95% CI: 3.206–6.575, P < 0.001) remained significantly associated with AMI.

## Discussion

This large-scale retrospective analysis of the NIS database provides contemporary insights into the incidence, associated factors, and clinical implications of in-hospital AMI among patients with obesity undergoing TAVR. Over the 7-year study period, we observed an overall AMI incidence of 2.5% among 19,971 patients with obesity undergoing TAVR, a rate consistent with previous reports [14]. Although obesity has traditionally been associated with a paradoxical protective effect—termed the “obesity paradox”—our findings suggest that this protection does not uniformly extend to all complications. Meta-analyses have demonstrated an obesity paradox in TAVR populations, typically showing lower all-cause mortality in patients with obesity compared with normal-weight counterparts [8]. However, this paradox has been increasingly challenged by evidence suggesting that the apparent survival benefit may reflect residual confounding from frailty and nutritional status rather than a direct protective effect of adiposity [9, 15]. Importantly, the obesity paradox has been primarily described for mortality and heart failure, whereas our study specifically examined AMI—a complication with distinct pathophysiology related to coronary obstruction [5]. Thus, our findings do not contradict the broader paradox literature but suggest that the protective effect, if real, may not uniformly extend to all in-hospital complications [9, 15].

### Comparative analysis between patients with obesity and those without obesity

Our comparative analysis revealed that, consistent with the obesity paradox [8, 16], patients with obesity had significantly lower in-hospital mortality (0.8% vs. 1.3%) than their counterparts without obesity, despite having a higher burden of comorbidities. However, despite these favorable overall outcomes, the incidence of AMI was comparable between the two groups (2.5% vs. 2.7%) [17]. This dissociation—obesity conferring a survival benefit but not an AMI benefit—suggests that the protective effect of obesity, if real, is complication-specific rather than universal [18].

Interestingly, patients with obesity had longer hospital stays (3 vs. 2 days) and higher hospitalization costs ($221,000 vs. $179,887) compared with patients without obesity. This may reflect a higher burden of post-procedural complications, including vascular access complications, heart failure exacerbation, and pulmonary complications, which have been shown to be more prevalent in obese patients undergoing TAVR [19]. These findings have important clinical and economic implications: the “obesity paradox” is not a blanket protection but a selective phenomenon—obesity improves survival but prolongs recovery and consumes more healthcare resources [8, 16, 19].

Crucially, the value of our study lies not in documenting an “acceptable” complication rate, but in demonstrating that the obesity paradox—well-described for mortality in TAVR populations—does not extend to AMI. By focusing specifically on patients with obesity and examining AMI as a distinct outcome, our study addresses an important knowledge gap. While prior investigations have predominantly examined composite endpoints or all-cause mortality, our analysis provides a detailed characterization of AMI in this high-risk population and identifies potentially modifiable associated factors—including anemia, electrolyte disorders, and weight loss—that have not been systematically explored in prior TAVR literature. These findings offer clinically actionable insights for preoperative risk stratification beyond the well-recognized association between obesity and cardiovascular risk.

### Patient demographics and the evolving understanding of obesity in TAVR

Our univariate analysis revealed a seemingly paradoxical finding: patients experiencing AMI were younger, more frequently belonging to the 18–64 age group in unadjusted analyses. This apparent contradiction may be explained by emerging evidence suggesting that nutritional and frailty status—rather than thinness *per se*—are the true determinants of adverse outcomes [15, 20]. Consistent with this perspective, the Trans-Pacific TAVR Registry demonstrated that underweight patients, despite being frailer, exhibited greater left ventricular mass regression at 1 year, challenging conventional BMI-centric risk assessment and shifting emphasis toward nutritional and frailty evaluations [9]. The younger age of AMI patients in our cohort may therefore reflect selection bias, wherein younger obese patients undergoing TAVR represent a subgroup with more aggressive disease phenotypes or complex pathology warranting earlier intervention [21]. This hypothesis is further supported by hemodynamic subtype research, which has shown that patients with paradoxical low-flow, low-gradient aortic stenosis—characterized by advanced disease despite preserved ejection fraction—experience the poorest outcomes in patients undergoing TAVR, suggesting that underlying hemodynamic profile, rather than age alone, is associated with post-procedural prognosis [22].

Beyond age-related findings, our unadjusted analysis revealed significant racial disparities, with Black and Hispanic patients disproportionately represented in the AMI group in univariate comparisons. These findings corroborate a comprehensive NIS database analysis documenting increased mortality in Hispanic patients and higher stroke risk in Black patients undergoing TAVR, with Hispanic women facing the highest mortality risk—highlighting the intersectionality of demographic risk factors [23]. The persistent underrepresentation of minority populations in TAVR clinical trials further underscores the need for equitable healthcare delivery [24]. Several interconnected mechanisms may explain these disparities, including differential access to specialized cardiovascular care, variations in comorbidity management, and socioeconomic factors that influence perioperative optimization [25].

### Hospital characteristics and system-level factors

The strong association between hospital characteristics and AMI risk warrants careful consideration. Patients treated in large hospitals had significantly higher odds of AMI compared to those in small hospitals. This finding, while counterintuitive at first glance, likely reflects complex case referral patterns to tertiary centers, where patients with severe comorbidities, complex valvular anatomy, or previous cardiac interventions are preferentially managed [26]. Elbadawi et al analyzed 72,123 patients across 400 US hospitals and demonstrated an inverse association between hospital procedural volumes and in-hospital mortality after aortic valve replacement [26]. However, the higher AMI risk observed in large centers may be attributable to their management of more complex cases, which is associated with elevated procedural risk despite superior resources and expertise [26]. Importantly, the NIS database lacks data on annual TAVR case volume at the hospital or operator level, which is a well-established predictor of outcomes [26]. Thus, our reliance on hospital bed size as a proxy for institutional capacity may not fully capture the impact of procedural experience on AMI risk. The observed association between large hospital size and AMI should be interpreted with this limitation in mind. The association between elective admission and lower AMI odds is consistent with evidence highlighting the potential benefits of optimal timing for intervention, which facilitates comprehensive preoperative assessment and optimization before acute decompensation occurs [27, 28].

### Preoperative comorbidities independently associated with in-hospital AMI among obese patients undergoing TAVR

Multivariable analysis identified several preoperative comorbidities independently associated with in-hospital AMI. Fluid and electrolyte disorders demonstrated the strongest association, a finding supported by large-scale evidence. A comprehensive NIS database analysis revealed that TAVR patients with concomitant hypo-osmolar hyponatremia had significantly increased risks of in-hospital mortality and adverse events, including AMI and cardiogenic shock [29]. These associations persisted after propensity score matching and sensitivity analysis, underscoring the critical role of electrolyte homeostasis in maintaining myocardial electrical stability and contractile function [29]. The vulnerability of this patient population is further illustrated by case reports of severe electrolyte disturbances—such as osmotic demyelination syndrome resulting from rapid sodium fluctuation—occurring post-procedurally [30]. Electrolyte imbalances may be associated with disruption of the delicate balance between myocardial oxygen demand and supply, which may be associated with ischemic events during the perioperative period characterized by significant fluid shifts and contrast administration [31].

In our analysis, anemia was identified using two distinct Elixhauser comorbidity categories: deficiency anemia and chronic blood loss anemia. Although these categories are derived from standardized ICD-10 coding definitions, we did not interpret them as mutually exclusive etiological subtypes. Rather, both were considered as overall markers of reduced hemoglobin status and comorbidity burden. Anemia emerged as a significant associated factor, with both deficiency anemia and chronic blood loss anemia independently associated with AMI. These findings align with research demonstrating the high prevalence and clinical impact of anemia in TAVR populations. Eitan et al evaluated changes in hemoglobin levels in TAVR patients and reported significant hemoglobin improvement and reduced anemia prevalence 5–12 months post-procedurally [32]. The authors concluded that gastrointestinal blood loss due to angiodysplasia—a feature of Heyde’s syndrome—is significantly reduced following TAVR, suggesting a potential link between correction of aortic stenosis and hemoglobin improvement [32]. This bidirectional relationship—anemia being associated with AMI, and TAVR potentially improving anemia—highlights the importance of considering preoperative hemoglobin status in risk assessment for patients with obesity undergoing TAVR [33].

Congestive heart failure emerged as a potent associated factor, consistent with the understanding that pre-existing myocardial dysfunction may be associated with increased susceptibility to ischemic events. Research on hemodynamic subtypes has demonstrated that patients with paradoxical low-flow, low-gradient aortic stenosis exhibit the highest mortality, poorest symptomatic benefit, and the least degree of reverse cardiac remodeling in patients undergoing TAVR among all subtypes, with this status independently predicting cardiovascular mortality [22]. These findings suggest that the specific hemodynamic profile, rather than simply the presence of heart failure, is a key determinant of post-procedural outcomes.

It is important to distinguish between obesity as a baseline characteristic and weight loss as a comorbidity. In this analysis, “weight loss” was identified using ICD-10-CM diagnosis codes and, consistent with the Elixhauser comorbidity classification [13], represents a marker of malnutrition, cachexia, or frailty rather than intentional weight reduction in patients with obesity. This distinction is critical because other neurological disorders and weight loss emerged as factors independently associated with AMI, likely reflecting overall frailty and comorbidity burden rather than direct mechanistic relationships. This interpretation aligns with the emerging consensus that frailty—not thinness *per se*—is more closely associated with adverse outcomes [15]. The editorial accompanying the Trans-Pacific TAVR Registry findings emphasizes that BMI is a crude anthropometric proxy that ignores sarcopenia, visceral adiposity, and unintentional weight loss—features that track more closely with resilience or frailty [15]. Thus, while our study used BMI-defined obesity as the inclusion criterion, the observed association between weight loss and AMI should be interpreted as a signal of underlying vulnerability rather than a paradoxical effect of obesity itself, and does not imply a direct causal pathway from weight loss to AMI. Similarly, peripheral vascular disorders and pulmonary circulation disorders reflect systemic atherosclerotic burden and right ventricular afterload, respectively. The Elixhauser category “pulmonary circulation disorders” is a broad administrative coding term that includes pulmonary hypertension and pulmonary embolism, among other conditions [13]. In our analysis, we did not interpret this as a specific pathophysiological diagnosis but rather as a general marker of right heart dysfunction and overall comorbidity burden, consistent with its intended use as an administrative risk-adjustment variable. Research comparing TAVR versus surgical aortic valve replacement (SAVR) in patients with pulmonary hypertension has demonstrated that the presence of pulmonary hypertension significantly impacts outcomes, with TAVR patients showing lower survival compared to SAVR patients in selected populations [34].

Similarly, diabetes mellitus did not retain statistical significance in our multivariable analysis. This lack of association may reflect the complex interplay between obesity, diabetes, and cardiovascular risk, or may be related to differences in diabetic severity and management not captured in administrative data [35, 36].

### In-hospital complications associated with AMI among obese TAVR hospitalizations

The strong independent associations between postoperative complications and AMI highlight important considerations for clinical management. Acute respiratory failure demonstrated the strongest association, which may reflect the interplay between respiratory and cardiac function. Hypoxemia from respiratory failure is associated with increased myocardial oxygen demand and reduced oxygen delivery, conditions that can coexist with myocardial ischemia [37]. Similarly, pulmonary edema and respiratory failure often occur together, suggesting a potential cycle of cardiopulmonary deterioration [38, 39].

Pneumonia and blood transfusion were also strongly and independently associated with AMI. Pneumonia is associated with systemic inflammation and increased metabolic demand, which may be linked to myocardial dysfunction and ischemic events [40]. The relationship between blood transfusion and AMI is complex and likely bidirectional. Anemia from procedural blood loss may necessitate transfusion while also reducing myocardial oxygen delivery, and both conditions are associated with ischemic risk [32]. Transfusion itself may be associated with increased blood viscosity and circulatory volume, elevating cardiac workload, and stored blood products can induce inflammatory responses that affect endothelial function and coagulation [32]. The association between anemia and outcomes in patients undergoing TAVR is further supported by evidence of post-procedural hematologic improvement. Studies have demonstrated that patients with baseline anemia show significant hemoglobin increases at 6 and 12 months following the procedure, suggesting a potential association between hemodynamic improvement from valve correction and amelioration of conditions predisposing to both anemia and transfusion need [32]. The reduction in gastrointestinal blood loss from angiodysplasia following valve replacement provides a mechanistic link between aortic stenosis correction and hematologic recovery [41].

Cardiac arrest is closely associated with severe myocardial ischemia, and both conditions often coexist in critically ill patients. Prolonged resuscitation efforts can themselves induce myocardial injury, further complicating the clinical picture [42]. The association between electrolyte disorders and cardiac arrest is supported by research demonstrating that electrolyte imbalances—particularly hypotonic hyponatremia—are associated with increased in-hospital mortality and adverse cardiovascular events [29].

### Clinical implications and integration with current evidence

Our findings should be contextualized within the evolving understanding of obesity in TAVR populations. The traditional “obesity paradox”—the notion that higher BMI confers a protective effect—is increasingly challenged by more nuanced analyses incorporating frailty, nutritional status, and body composition [9, 22]. The Trans-Pacific TAVR Registry highlighted that the absence of direct frailty measures leaves the inherent limitations of BMI unaddressed, a caveat that equally applies to our study [9]. As the accompanying editorial emphasized, clinicians should shift focus from BMI-based triage toward actionable parameters—such as albumin, hemoglobin, and quadriceps circumference—that more directly correlate with functional recovery [15].

These findings carry several clinical implications. First, the identification of multiple preoperative factors associated with AMI presents opportunities for targeted optimization. Given that anemia affects up to 67.5% of TAVR candidates and demonstrates significant post-procedural improvement [32], its role as a marker of increased risk warrants attention in preoperative assessment. Second, the strong association between elective admission underscores the importance of timely TAVR referral—before acute decompensation occurs—to allow for comprehensive preoperative assessment and optimization of the patient’s condition. Third, the observed racial disparities highlight the need for targeted interventions to ensure equitable access to high-quality cardiovascular care, particularly in light of well-documented outcome disparities among minority populations undergoing TAVR [23]. Fourth, clinicians should move beyond simplistic BMI-based risk stratification toward a more comprehensive evaluation of modifiable resilience markers, including nutritional status, hemoglobin levels, and functional capacity [9, 15, 22].

### Limitations

This study has several limitations inherent to its retrospective design and administrative database source. First, the NIS database captures only in-hospital events, precluding assessment of post-discharge AMI occurrences and long-term outcomes. Second, coding inaccuracies in ICD-10-CM diagnoses may lead to misclassification bias, although previous validation studies have demonstrated reasonable accuracy for major cardiovascular events. Our definition of AMI relied exclusively on ICD-10-CM diagnostic codes, without clinical adjudication or differentiation between ST-segment elevation myocardial infarction (STEMI) and non-ST-segment elevation myocardial infarction (NSTEMI). This approach captures all AMI types (STEMI, NSTEMI, and unspecified) as a composite outcome, which may introduce heterogeneity and limit the granularity of our findings, as STEMI and NSTEMI differ in their pathophysiology, management, and prognostic implications. The absence of electrocardiographic, biomarker, and angiographic data precludes further subclassification and assessment of AMI severity or mechanism. Additionally, the NIS database does not provide the exact timing of AMI relative to the TAVR procedure, and administrative coding does not reliably establish event chronology. Consequently, AMI was defined as an in-hospital diagnosis during the index hospitalization and may include events occurring before, during, or after the TAVR procedure, precluding distinction between periprocedural and spontaneous AMI. Third, the database lacks granular clinical information including angiographic data, details of the culprit vessel, coronary anatomy, specific TAVR procedural details (e.g., valve type/platform, approach, concomitant procedures), perioperative medications (antiplatelet therapy, anticoagulation), surgical risk scores (e.g., STS-PROM, EuroSCORE II) and laboratory values (troponin levels, hemoglobin, electrolytes), as well as annual hospital and operator TAVR procedure volume, and patient-level cardiovascular risk factors such as smoking status and family history of cardiovascular disease, limiting our ability to adjust for these potential confounders. The absence of valve-specific data precludes comparative analysis of outcomes across different transcatheter heart valve platforms, which may have varying profiles for coronary obstruction and other procedural complications. The absence of procedure volume data is particularly relevant given the established volume-outcome relationship in TAVR. Additionally, the lack of angiographic and coronary anatomical data precludes assessment of the relationship between pre-existing coronary artery disease, the mechanism of AMI (e.g., coronary obstruction vs. embolic events), and post-procedural outcomes. Fourth, the observational design precludes causal inference; the associations identified between comorbidities, complications, and AMI should be interpreted as associations rather than causal relationships. Fifth, unmeasured confounding—including frailty measures (grip strength, gait speed), socioeconomic factors, and institutional protocols—may influence the observed relationships. The Trans-Pacific TAVR Registry’s finding that lack of direct frailty measures leaves BMI’s known limitations unaddressed applies equally to our study [9]. Sixth, our study focused exclusively on obese patients, limiting generalizability to normal-weight TAVR populations, although this specificity was intentional to address the unique considerations in this growing patient population. Finally, the lack of echocardiographic follow-up data, as noted in other studies, limits our ability to assess cardiac remodeling and its relationship to outcomes.

### Conclusions

In this national analysis of patients with obesity undergoing TAVR, in-hospital AMI occurred in 2.5%, a rate comparable to that observed in patients without obesity (2.7%, P = 0.295). Despite lower overall mortality in the obesity cohort (0.8% vs. 1.3%, P = 0.002)—consistent with the obesity paradox—AMI risk was not similarly attenuated, suggesting that the protective effect of obesity does not uniformly extend to all periprocedural complications. Rather than simply documenting an expected complication rate, the clinical value of our study lies in identifying potentially modifiable factors associated with AMI specifically in the obesity cohort—including anemia, fluid and electrolyte disorders, and weight loss—that may inform targeted preoperative optimization. These findings may help refine perioperative risk assessment, although prospective studies with more granular clinical data are needed to validate these associations and clarify event chronology.

## Supplementary Material

Suppl 1 ICD-10 codes used for patient identification.

## Data Availability

The data that support the findings of this study are publicly available from the Healthcare Cost and Utilization Project (HCUP) Central Distributor. The National Inpatient Sample (NIS) database official information page is: https://hcup-us.ahrq.gov/nisoverview.jsp. The dataset can be accessed via the AHRQ HCUP Central Distributor: https://hcup-us.ahrq.gov/tech_assist/centdist.jsp. Researchers must complete a data use agreement to obtain the data. The authors did not have any special access privileges.

## Abbreviations

AHRQ: Agency for Healthcare Research and Quality
AIDS: acquired immunodeficiency syndrome
AMI: acute myocardial infarction
BMI: body mass index
CI: confidence interval
HCUP: Healthcare Cost and Utilization Project
ICD-10-CM: International Classification of Diseases, Tenth Revision, Clinical Modification
ICD-10-PCS: International Classification of Diseases, Tenth Revision, Procedure Coding System
ICD-CM: International Classification of Diseases, Clinical Modification
IRB: Institutional Review Board
LOS: length of stay
NIS: National Inpatient Sample
OR: odds ratio
SAVR: surgical aortic valve replacement
TAVR: transcatheter aortic valve replacement
TOTCHG: Total Charges
VARC: Valve Academic Research Consortium
