# Supplementary material for: Incidence and Factors Associated With In-Hospital Acute Myocardial Infarction in Patients With Obesity Undergoing Transcatheter Aortic Valve Replacement: A National Inpatient Sample Analysis
Source: Cardiol Res. 2026 Aug 31;17(5):476–88. doi: 10.14740/cr2257 (PMC13568734; doi:10.14740/cr2257)
Supplement: Supplementary file 1 [file cr-17-05-476-s001.docx]

**Suppl 1. ICD-10 Codes Used for Patient Identification**

| **Category** | **ICD Codes** | **Description** | **Validation Reference(s)** |
| --- | --- | --- | --- |
| **TAVR Procedure Codes** | 02RF38H, 02RF38Z, 02RF3JH, 02RF3KH, 02RF37H, 02RF37Z, 02RF3JZ, and 02RF3KZ | Transcatheter aortic valve replacement | [1][2] |
| **AMI Diagnosis Codes** | I21.0, I21.01, I21.02, I21.09, I21.1, I21.11, I21.19, I21.2, I21.21, I21.29, I21.3, I21.4, I21.9, I21.A, I21.A1, I21.A9, I22.0, I22.1, I22.2, I22.8, and I22.9 | Acute myocardial infarction | [3] |
| **Obesity Diagnosis Codes** | E66, Z68.30, Z68.31, Z68.32, Z68.33, Z68.34, Z68.35, Z68.36, Z68.37, Z68.38, Z68.39Z68.41, Z68.42, Z68.43, Z68.44, and Z68.45 | Body mass index ≥30 kg/m² | [4][5] |

**Reference(s)**

[1]Alsughayer A, et al. Body Mass Index and Outcomes of Transcatheter Aortic Valve Replacement: A Retrospective Observational Study. Curr Probl Cardiol. 2023;48(10):101879. doi:10.1016/j.cpcardiol.2023.101879

[2]National Institutes of Health. Table E4: Procedure Codes for Aortic Valve Reinterventions. In: PMC9801294. Available from: <https://pmc.ncbi.nlm.nih.gov/articles/PMC9801294/table/tblE4/>

[3]Slezak, J., et al., Validation of ICD‐10 Diagnosis Codes for Identification of Acute Myocardial Infarction From a US Integrated Healthcare System. Pharmacoepidemiology and Drug Safety, 2025. 34(7).

[4]Ammann, E.M., et al., Assessment of obesity prevalence and validity of obesity diagnoses coded in claims data for selected surgical populations. Medicine, 2019. 98(29).

[5]Ammann, E.M., et al., Validation of body mass index (BMI)‐related ICD‐9‐CM and ICD‐10‐CM administrative diagnosis codes recorded in US claims data. Pharmacoepidemiology and Drug Safety, 2018. 27(10): p. 1092-1100.
